# A dual-factor model perspective on depressed inpatients: examining the dynamics of mental health and therapy outcomes

**DOI:** 10.3389/fpsyt.2023.1295032

**Published:** 2024-01-11

**Authors:** Julia Brailovskaia, Ruth von Brachel, Franziska van Hall, Tobias Teismann, Gerrit Hirschfeld, Jürgen Margraf

**Affiliations:** ^1^Mental Health Research and Treatment Center, Ruhr-University Bochum, Bochum, Germany; ^2^DZPG (German Center for Mental Health), Partner Site Bochum/Marburg, Bochum, Germany; ^3^CuraMed Private Clinic Stillachhaus, Oberstdorf, Germany; ^4^University of Applied Sciences, Bielefeld, Germany

**Keywords:** depression, anxiety, positive mental health, inpatient treatment, dual-factor model

## Abstract

**Background:**

The dual-factor model of mental health posits that mental health and mental illness constitute two distinct axes; accordingly the model identifies four mental health groups: (1) complete mental health, (2) troubled, (3) vulnerable, (4) symptomatic but content. Yet, only a few studies investigated effectiveness of therapy on both dimensions of mental health simultaneously. Against this background, the present study aimed to determine proportions and changes of group assignments in depressed inpatients undergoing therapy.

**Method:**

*N* = 1,044 depressed inpatients (age in years: M = 53.36, SD = 9.81, range: 17–83) completed a pre- and a post-treatment survey including questionnaires on depression, anxiety, and positive mental health. A total of *n* = 328 persons completed the survey also at 6-month and 12-month follow-up assessments.

**Results:**

In the classification that included depression symptoms and positive mental health, 49% of the participants were classified as troubled and 13.2% were classified as completely mentally healthy at the pre-treatment assessment. At the post-treatment, 9.5% were classified as troubled and 55.7% were classified as completely mentally healthy. In the classification that included anxiety symptoms and positive mental health, 21.9% of the participants were classified as troubled and 14.2% were classified as completely mentally healthy at the pre-treatment assessment. At the post-treatment, 3.7% were classified as troubled and 56.1% were classified as completely mentally healthy. About 10 to 20% of patients showed an improvement in depression/anxiety *and* positive mental health, whereas another 10 to 20% showed a reduction in depression/anxiety, but only a minor increase in positive mental health between pre- and post-treatment.

**Conclusion:**

Findings are in line with past research inspired by the dual-factor model in showing that enhancing positive mental health and alleviating psychopathology do not always co-occur in treatment. It is therefore important to implement measures of both psychopathology and positive mental health in therapy outcome studies, and to promote interventions targeting both psychopathology and positive mental health.

## 1 Introduction

In addition to the absence of mental disorder, positive mental health has been recognized as a key element of well-being (1, 2): to exhibit *complete mental health*, an individual should not experience psychopathology, and additionally exhibit high levels of subjective and psychological well-being (3). Whereas in the past, clinical psychology was primarily concerned with eliminating or reducing psychopathology, with the rise of positive psychology the focus is increasingly on the positive dimensions of health (4–9). In addition to negative variables such as symptoms and risk factors of mental health, recent attention has focused on positive factors of mental health such as optimism, life satisfaction, happiness, self-acceptance, purpose in life and self-efficacy, because they significantly influence the course of mental disorders (10–15). Several studies found positive mental health, i.e., subjective and psychological well-being (1, 16), to be a particularly important predictor of remission of mental disorders, such as specific phobia, social phobia, panic disorder and agoraphobia (12, 17–19) as well as suicidality (20). Still, in psychotherapy research a focus on reducing psychopathology continues to prevail. In this sense, symptom reduction, remission and response rates are considered central markers of treatment success (21), while improvements in positive mental health are often used only as secondary outcome measures (22).

The relationship between positive and negative mental health has been conceptualized in different ways: the categorical view of healthy and disordered as two qualitatively different categories corresponds to the traditional medical model (23). Also unidimensional approaches to psychopathology implicitly assume that health and illness represent different manifestations on a single dimension [cf., (24, 25)], whereas multidimensional models assume that health and disorder are two distinct dimensions (1, 3, 6, 22, 26–29). Such two-dimensional models, called “dual-factor models,” postulate a second factor, which includes positive mental health or minimal/maximal well-being, in addition to a disorder factor that ranges from minimal to maximal complaints or symptoms (1); accordingly these models allow to differentiate between four mental health groups (3): *complete mental health* (low psychopathology and high positive mental health), *vulnerable* (low psychopathology and low positive mental health), *troubled* (high psychopathology and low positive mental health) as well as *symptomatic but content* (high psychopathology and high positive mental health). Dual-factor models of mental health suggests that the absence of mental illness does not equal the presence of mental health (1), and that enhancing positive mental health and alleviating psychopathology do not automatically go hand-in-hand (22). In support of the dual-factor model, a wide range of studies has shown that positive mental health and psychopathology form two negatively related – but not identical – dimensions of mental health [e.g., (1, 13, 29)], and that both can be present at the same time [e.g., (3, 30, 31)]. Furthermore, there are many studies showing that various treatments are effective both in increasing positive mental health and alleviating psychopathology symptoms [e.g., (32–35)]. Yet, only few studies investigated the interrelatedness of therapeutic effects on both dimensions of mental health – psychopathology and positive mental health – at the same time (22, 36). In a seminal study, Trompetter, Lamers (22) were able to show that about 36% of patients improved on both positive mental health and depression symptoms in the course of a self-help intervention and that 64% improved only on either one of the outcomes but not the other. In a similar vein, van Agteren, Ali (36) found that 71% of participants joining an online, group-based mental health intervention, improved in both mental well-being and depression symptoms, whereas 14% improved either in well-being only or in distress symptoms only. These findings support the dual-factor model and suggest that it is important to systematically implement measures of both psychopathology and positive mental health in mental health care and therapy evaluations. However, Trompetter, Lamers (22) and van Agteren, Ali (36) investigated rather specific interventions and did not consider the four possible categories of the dual-factor model.

In order to address this lack in the scientific literature, the present study aimed to determine the proportion of depressed inpatients belonging to the four categories of the dual-factor model pre-treatment and post-treatment as well as three to 6 months after treatment. Of particular interest here is the extent to which positive mental health and psychopathology, i.e., depression and anxiety symptoms, respond independently to treatment. Possible differential effects would not only support the requirement to routinely assess both markers of positive and negative mental health in treatment studies (8), but also to complement categorical markers of treatment outcome (i.e., response and remission rates) with a more comprehensive marker of treatment outcome: complete mental health [cf., (37)].

## 2 Materials and methods

### 2.1 Procedure

Data was collected between 25th of July and 14th of November 2022 in the CuraMed Private Clinic Stillachhaus – an inpatient clinic specialized in the treatment of depression in a rural area in the south of Germany. All inpatients were invited to take part in the study and to fill out the questionnaires in an online survey within the first 2 days of their stay (pre-treatment, T1) and again 1 day before discharge (post-treatment, T2). There were no specific inclusion or exclusion criteria for participation in the present study. Six months (6-month follow-up, T3) and 12 months (12-month follow-up, T4) after discharge, they were again invited to take part in the online survey via email by a team of medical assistants in the private clinic and were reminded via email twice. To ensure data protection, no information which could possibly lead to identification was assessed in the online study. All personal information (e.g., date of birth, diagnoses, marital status, medication) was assessed by the clinicians in the treatment center and later collected from the patient files. The local ethics committee at the Faculty of Psychology of the Ruhr-University Bochum approved the implementation of the study (2017/387), and the study was registered at clinical trials.

### 2.2 Treatment

Patients received an integrative multimodal psychosomatic treatment approach consisting of at least two up to three weekly individual therapy sessions with a licensed psychotherapist and three up to six sessions of group therapy. Group services include resilience training, mindfulness-based-therapy, stress awareness group, schema therapy group (38), and metacognitive therapy (MCT) group (39). Accompanying common relaxation techniques, sports, and movement therapy groups were offered. In accordance with the patient’s preferences, antidepressant medication was used as indicated for moderate to severe depression according to the national guidelines (40). Mainly antidepressants, e.g., Selective Serotonin Reuptake Inhibitors (Sertraline, (Es)-Citalopram, Fluoxetine), Selective Serotonin and Noradrenalin Reuptake Inhibitors (Venlafaxine, Duloxetine, Milnacipran), Noradrenaline and Specific Serotonergic Antidepressants (Mirtazapine), and Norepinephrine Dopamine Reuptake Inhibitors (Bupropion), were used in therapeutic doses (see Table 1). Drug treatment was closely monitored by a specialist over the course of treatment, and regularly monitored by laboratory chemistry (including therapeutic drug monitoring) according to the official guidelines.

**Table 1:** Demographic and further statistics of the investigated sample.

	*N* = 1,044	*n* = 328
	% (*n*)	% (*n*)
Gender		
Women	54.1 (565)	55.8 (183)
Men	45.9 (479)	44.2 (145)
Marital Status		
Single	69.3 (724)	72.6 (238)
With Partner	30.7 (320)	27.4 (90)
Highest Education Level		
University	65.3 (682)	68.9 (226)
Not University	34.7 (362)	31.1 (102)
Employment Status		
Employee	82.7 (863)	86.3 (283)
Unemployed/Retired	17.3 (181)	13.7 (45)
Main Diagnosis		
Recurrent Depressive Disorder	56.4 (589)	57.6 (189)
Depressive Episode	35.2 (368)	34.1 (112)
Phobic Anxiety Disorders	2.8 (29)	4.0 (13)
Bipolar Affective Disorder	1.3 (14)	0.3 (1)
Post-Traumatic Stress Disorder	1.1 (11)	0.3 (1)
Schizoaffective Disorder (depressive Type)	0.5 (5)	0.3 (1)
Other (e.g., Eating Disorders)	2.7 (28)	3.4 (11)
Further Diagnosis		
Somatoform Disorders	19.3 (201)	18.9 (62)
Adjustment Disorders	5.5 (57)	4.6 (15)
Other Anxiety Disorders (Panic Disorder, Generalized Anxiety Disorder)	5.3 (55)	5.8 (19)
Nonorganic Sleep Disorders	5.2 (54)	4.6 (15)
Phobic Anxiety Disorders	2.1 (22)	2.7 (9)
Recurrent Depressive Disorder	1.6 (17)	0.9 (3)
Depressive Episode	1.1 (12)	2.7 (9)
Bipolar Affective Disorder	0.3 (3)	0
Other (e.g., Eating Disorders)	2.8 (29)	2.7 (9)
None	56.9 (594)	57.0 (187)
Medication Pre-Treatment		
Antidepressants	41.6 (434)	41.2 (135)
Antipsychotic	6.6 (69)	5.5 (18)
Sedatives	4.0 (42)	3.7 (12)
Medication Post-Treatment		
Antidepressants	47.7 (498)	46.0 (151)
Antipsychotic	9.2 (96)	6.7 (22)
Sedatives	1.4 (15)	1.2 (4)

Considering gender, no diverse persons participated in the study.

### 2.3 Participants

Overall, 1,247 patients completed the pre-treatment survey. Of them 1,044 persons (age in years: M = 53.36, SD = 9.81, range: 17–83) completed also the post-treatment survey and, therefore, were included in the present analyses. A total of *n* = 328 persons (age in years: M = 52.45, SD = 9.35, range: 19–78) completed the survey also at 6-month and 12-month follow-ups. Table 1 shows the demographic statistics of *N* = 1,044 and *n* = 328. A multivariate analysis of variance (MANOVA) revealed no significant differences between participants who completed only the pre-treatment survey and those who completed the pre- and post-treatment survey regarding demographic variables, depression symptoms, anxiety symptoms, positive mental health, medical treatment at pre-treatment. A second MANOVA showed that participants who completed all four surveys were significantly younger, *F*(1, 1,042) = 4.135, *p* = 0.042, effect-size: partial eta-squared (*η*^2^_p_) = 0.004 (small effect), and had significantly lower post-treatment depression symptoms, *F*(1, 1,042) = 9.566, *p* = 0.002, *η*^2^_p_ = 0.009 (small effect), than participants who completed only the pre- and post-treatment surveys. Other demographic variables, anxiety symptoms and positive mental health did not significantly differ between both groups at pre-treatment.

### 2.4 Measures

*Depression Anxiety Stress Scales 21 – Depression and Anxiety Subscales* [DASS-21; original version: 41, German language version: 42]. The DASS-21 was used to assess symptoms of depression and anxiety over the past week. Both subscales comprise seven items (e.g., depression subscale: “I felt that life was meaningless”; anxiety subscale: “I felt scared without any good reason”). All items are rated on a 4-point Likert-type scale (0 = *did not apply to me at all*, 3 = *applies to me very much or most of the time*). Higher sum scores indicate more severe symptoms. The total sum score of each subscale can range from zero to 21. Following previous research (42), scores ≥10 are considered as a problematic level of depression symptoms, and scores below 10 are considered as a non-problematic level of depression symptoms; for anxiety symptoms, scores ≥6 are considered as problematic and scores below 6 are considered as non-problematic. Scale reliability for the pre- and post-treatment (*N* = 1,044) of depression symptoms was Cronbach’s *α* = 0.878 and 0.888, and of anxiety symptoms it was *α* = 0.785 and 0.781.

*Positive Mental Health Scale* [PMH-Scale; original German language version: 16]. The PMH-Scale is a well-established instrument for the measurement of emotional, cognitive, social and psychological well-being. It consists of nine items that are rated on a 4-point Likert-type scale (e.g., “I enjoy my life”; 0 = *do not agree*, 3 = *agree*). Higher sum scores indicate higher levels of positive mental health. The total sum score can range from 0 to 27. Following previous research (30, 31), scores ≥14 are considered as moderate to high levels of positive mental health and scores below 14 are considered as low positive mental health. Scale reliability for the pre- and post-treatment (*N* = 1,044) of positive mental health was *α* = 0.877 and 0.933.

Both instruments were used at all measurement time points.

### 2.5 Statistical analyses

Statistical analyses were conducted with the Statistical Package for the Social Sciences (SPSS 28). After descriptive analyses, we ran repeated measure analyses of variance (ANOVAs; within factor-design) to test potential changes of the investigated variables between pre- and post-treatment for *N* = 1,044. For all variables, the assumption of sphericity (Mauchly’s test) was violated. Thus, we applied the Greenhouse–Geisser correction (*ε*). We used *η*^2^_p_ as the effect-size measure.

Then, following available literature (30, 31, 42), for each symptom of negative mental health, participants were classified into four mental health groups based on the problematic or non-problematic levels of depression symptoms (scores <10 vs. scores ≥10) or anxiety symptoms (scores <6 vs. scores ≥6), and low versus moderate to high levels of positive mental health (scores <14 vs. scores ≥14) (*N* = 1,044). Thus, the classification for depression symptoms was: *complete mental health* (depression symptoms score < 10 and positive mental health score ≥ 14), *vulnerable* (depression symptoms score < 10 and positive mental health score < 14), *troubled* (depression symptoms score ≥ 10 and positive mental health score < 14), and *symptomatic but content* (depression symptoms score ≥ 10 and positive mental health score ≥ 14); for anxiety symptoms it was: *complete mental health* (anxiety symptoms score < 6 and positive mental health score ≥ 14), *vulnerable* (anxiety symptoms score < 6 and positive mental health score < 14), *troubled* (anxiety symptoms score ≥ 6 and positive mental health score < 14) and *symptomatic but content* (anxiety symptoms score ≥ 6 and positive mental health score ≥ 14). The classification was conducted for the pre- and the post-treatment data assessment. After the classification, we counted the number of participants in each mental health group at both time points, as well as how many participants changed the group between pre- and the post-treatment to assess the group fluctuation.

To assess the stability of the treatment effect, we ran repeated measure ANOVAs (within factor-design) to test potential changes of the investigated variables between post-treatment and both follow-ups for *n* = 328. For all variables, the assumption of sphericity (Mauchly’s test) was violated. Thus, we applied the Greenhouse–Geisser correction (*ε*). We used *η*^2^_p_ as the effect-size measure of main effects (measurement time point), and Cohen’s *d*_Repeated Measures_ as effect-size measure of post-hoc comparisons. All post-hoc comparisons were Bonferroni-corrected (level of significance: *p* < 0.05, two-tailed).

Then, we conducted the classification for post-treatment, 6-month follow-up and 12-month follow-up data assessment for *n* = 328. After the classification, we counted the number of participants in each mental health group at the three time points, as well as how many participants changed the group between post-treatment and 6-month follow-up as well as between 6-month follow-up and 12-month follow-up data to assess the group fluctuation.

## 3 Results

### 3.1 Treatment effect: pre-treatment to post-treatment

The ANOVA showed that depression symptoms at pre-treatment (M = 9.76, SD = 5.13, range: 0–21) were significantly higher than depression symptoms at post-treatment (M = 4.06, SD = 4.01, range: 0–21), *F*(1, 1,043) = 1467.787, *p* < 0.001, *η*^2^_p_ = 0.585 (large effect). Also, anxiety symptoms were significantly higher at pre-treatment (M = 6.44, SD = 4.41, range: 0–21) than at post-treatment (M = 3.15, SD = 3.16, range: 0–21), *F*(1, 1,043) = 797.572, *p* < 0.001, *η*^2^_p_ = 0.433. In contrast, positive mental health was significantly lower at pre-treatment (M = 7.97, SD = 5.08, range: 0–26) than at post-treatment (M = 14.50, SD = 6.11, range: 0–27), *F*(1, 1,043) = 1328.900, *p* < 0.001, *η*^2^_p_ = 0.560 (large effect). Thus, there was a remarkable decrease of depression and anxiety symptoms between pre- and post-treatment and a remarkable increase of positive mental health.

**Figure 1:**
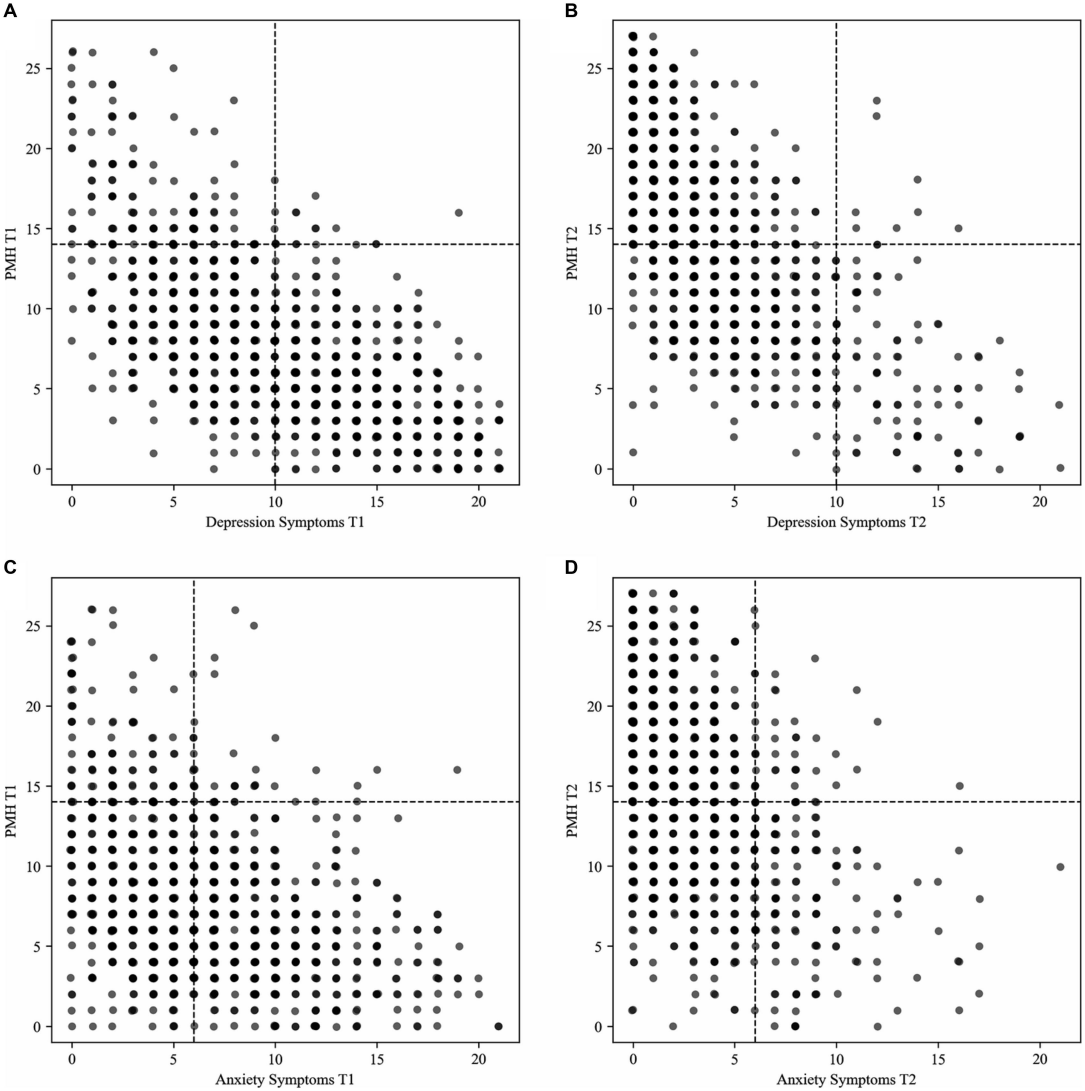
Distribution of the sample according to the mental health groups for the classification **(A)** depression symptoms and positive mental health pre-treatment; **(B)** depression symptoms and positive mental health post-treatment; **(C)** anxiety symptoms and positive mental health pre-treatment; **(D)** anxiety symptoms and positive mental health post-treatment. *N* = 1,044; T1, pre-treatment; T2, post-treatment; PMH, positive mental health.

Table 2 and Figure 1 show how many participants were assigned to the four mental health groups at pre- and post-treatment.

**Table 2:** Distribution of the sample according to the mental health groups (pre- and post-treatment).

	T1	T2
	% (*n*)	% (*n*)
Depression Symptoms vs. Positive Mental Health		
Complete Mental Health	13.2 (138)	55.7 (582)
Vulnerable	35.9 (375)	33.8 (353)
Troubled	49.0 (512)	9.5 (99)
Symptomatic but Content	1.8 (19)	1.0 (10)
Anxiety Symptoms vs. Positive Mental Health		
Complete Mental Health	14.2 (148)	56.1 (586)
Vulnerable	63.0 (658)	39.6 (413)
Troubled	21.9 (229)	3.7 (39)
Symptomatic but Content	0.9 (9)	0.6 (6)

*N* = 1,044; T1, pre-treatment; T2, post-treatment.

In the classification that included depression symptoms and positive mental health, most participants were assigned to the group *troubled* at pre-treatment, and to the group *complete mental health* at post-treatment (see Table 2 and Figure 1). In the classification that included anxiety symptoms and positive mental health, most participants were assigned to the group *vulnerable* at pre-treatment, and to the group *complete mental health* at post-treatment (see Table 2 and Figure 1).

Table 3 shows how many participants changed classification assignment from pre- to post-treatment. In the classification that included depression symptoms and positive mental health, 66.5% of the participants changed classification assignment. Most assignments changed to the group *complete mental health* (43.4%) (see Table 3). In the classification that included anxiety symptoms and positive mental health, 55.4% of the participants changed classification assignment. Again, most assignments changed to the group *complete mental health* (43.0%) (see Table 3).

**Table 3:** Changes between the mental health groups (pre- to post-treatment data assessment).

	T1 ➔ T2
	% (*n*)
Depression Symptoms vs. Positive Mental Health	
Complete Mental Health ➔ Troubled	0
Complete Mental Health ➔ Vulnerable	0.8 (8)
Complete Mental Health ➔ Symptomatic but Content	0.1 (1)
Vulnerable ➔ Complete Mental Health	21.2 (221)
Vulnerable ➔ Troubled	1.3 (14)
Vulnerable ➔ Symptomatic but Content	0.3 (3)
Troubled ➔ Complete Mental Health	20.9 (218)
Troubled ➔ Vulnerable	19.7 (206)
Troubled ➔ Symptomatic but Content	0.5 (5)
Symptomatic but Content ➔ Complete Mental Health	1.3 (14)
Symptomatic but Content ➔ Troubled	0.2 (2)
Symptomatic but Content ➔ Vulnerable	0.2 (2)
No Changes	33.5 (350)
Anxiety Symptoms vs. Positive Mental Health	
Complete Mental Health ➔ Troubled	0
Complete Mental Health ➔ Vulnerable	1.1 (11)
Complete Mental Health ➔ Symptomatic but Content	0
Vulnerable ➔ Complete Mental Health	33.4 (349)
Vulnerable ➔ Troubled	0.9 (9)
Vulnerable ➔ Symptomatic but Content	0.2 (2)
Troubled ➔ Complete Mental Health	8.9 (93)
Troubled ➔ Vulnerable	9.9 (103)
Troubled ➔ Symptomatic but Content	0.3 (3)
Symptomatic but Content ➔ Complete Mental Health	0.7 (7)
Symptomatic but Content ➔ Troubled	0
Symptomatic but Content ➔ Vulnerable	0.1 (1)
No Changes	44.6 (466)

*N* = 1,044; T1, pre-treatment; T2, post-treatment.

### 3.2 Stability of treatment effects: post-treatment, 6- and 12-month follow-up

Table 4 shows descriptive statistics (post-treatment to 12-month follow-up) of the subsample (*n* = 328) that responded to all surveys and their scale reliability.

**Table 4:** Descriptive statistics and scale reliability of the investigated variables for the subsample (*n* = 328; T2 to T4).

	T2		T3		T4	
	M(SD)	*α*	M(SD)	*α*	M(SD)	*α*
Depression Symptoms	3.49 (3.26)	0.843	4.93 (4.33)	0.906	4.79 (4.54)	0.903
Anxiety Symptoms	3.01 (2.85)	0.743	3.03 (3.11)	0.768	3.11 (3.46)	0.811
Positive Mental Health	14.93 (5.62)	0.919	13.50 (6.49)	0.945	14.00 (6.79)	0.950

T2 to T4, measurement time points; M, mean; SD, standard deviation; *α*, Cronbach’s *α*.

For depression symptoms, the ANOVA revealed a significant main effect for measurement time point, *F*(1.915, 626.183) = 24.627, *p* < 0.001, *η*^2^_p_ = 0.070. Pairwise comparisons showed that depression symptoms were significantly lower at post-treatment than at 6-month follow-up (*d*_Repeated Measures_ = 0.427, small effect) and 12-month follow-up (*d*_Repeated Measures_ = 0.359, small effect). For anxiety symptoms, the ANOVA provided no significant differences between the time points, *F*(1.901, 621.586) = 0.214, *p* = 0.796. For positive mental health, the ANOVA revealed a significant main effect for measurement time point, *F*(1.802, 589.381) = 11.552, *p* < 0.001, *η*^2^_p_ = 0.034. Pairwise comparisons showed that positive mental health was significantly higher at post-treatment than at 6-month follow-up (*d*_Repeated Measures_ = 0.281, small effect) and 12-month follow-up (*d*_Repeated Measures_ = 0.169, small effect).

**Figure 2:**
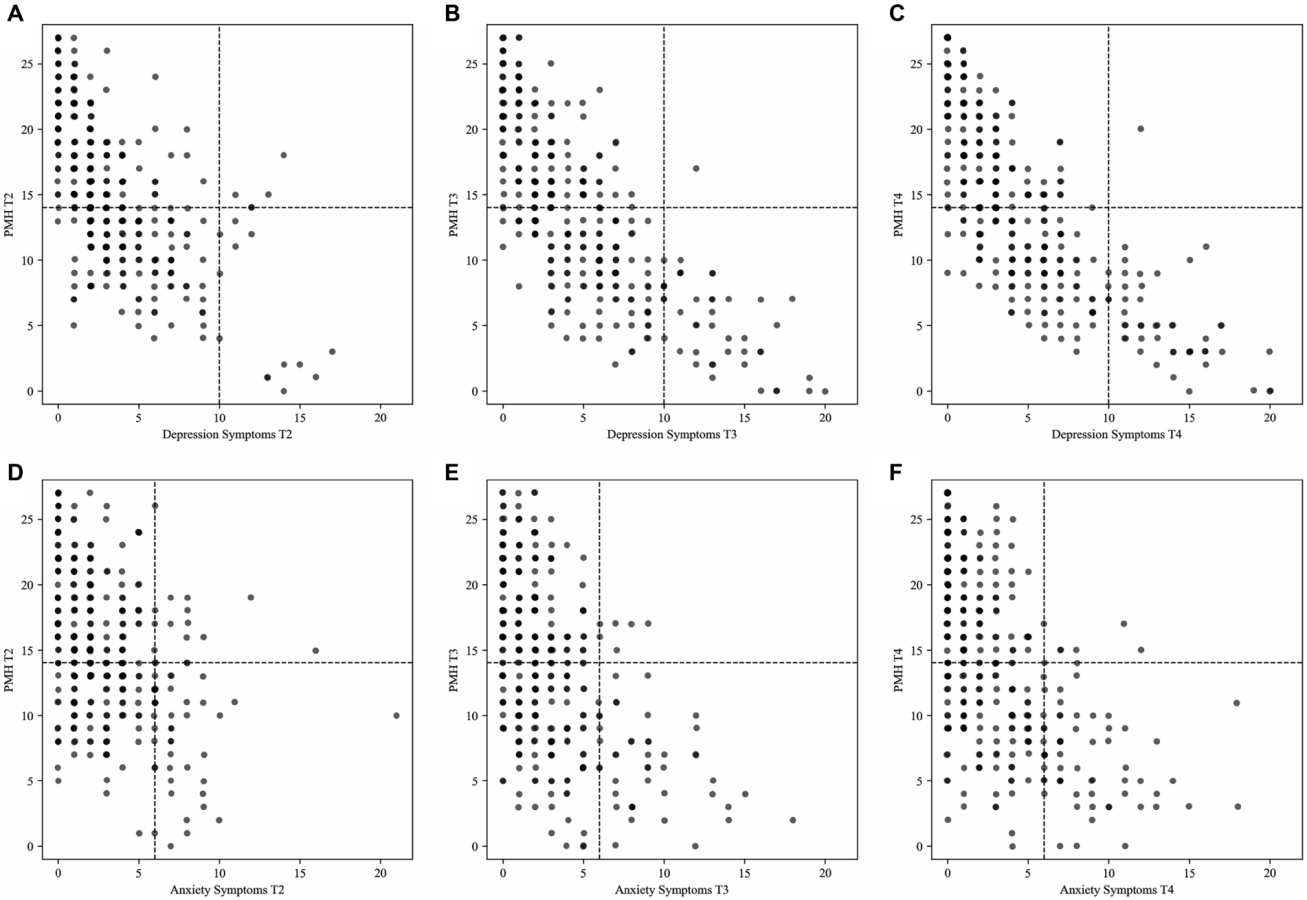
Distribution of the subsample (*n* = 328) according to the mental health groups for the classification **(A)** depression symptoms and positive mental health post-treatment; **(B)** depression symptoms and positive mental health 6-month follow-up; **(C)** depression symptoms and positive mental health 12-month follow-up; **(D)** anxiety symptoms and positive mental health post-treatment; **(E)** anxiety symptoms and positive mental health 6-month follow-up; **(F)** anxiety symptoms and positive mental health 12-month follow-up. T2, post-treatment; T3, 6-month follow-up; T4, 12-month follow-up; PMH, positive mental health.

Table 5; Figure 2 show how many participants were assigned to the four mental health groups at post-treatment, 6-month follow-up and 12-month follow-up.

**Table 5:** Distribution of the sample according to the mental health groups for the subsample (*n* = 328; T2 to T4).

	T2	T3	T4
	% (*n*)	% (*n*)	% (*n*)
Depression Symptoms vs. Positive Mental Health			
Complete Mental Health	57.3 (188)	50.3 (165)	51.8 (170)
Vulnerable	37.2 (122)	36.0 (118)	33.2 (109)
Troubled	4.0 (13)	13.4 (44)	14.6 (48)
Symptomatic but Content	1.5 (5)	0.3 (1)	0.3 (1)
Anxiety Symptoms vs. Positive Mental Health			
Complete Mental Health	58.2 (191)	50.6 (166)	51.5 (169)
Vulnerable	39.9 (131)	44.8 (147)	42.1 (138)
Troubled	1.2 (4)	4.6 (15)	5.8 (19)
Symptomatic but Content	0.6 (2)	0	0.6 (2)

T2 to T4, measurement time points.

In the classification that included depression symptoms and positive mental health as well as in the classification that included anxiety symptoms and positive mental health, most participants were assigned to the group *complete mental health* at all three time points (see Table 5; Figure 2). Table 6 shows how many participants changed classification assignment from post-treatment to 6-month follow-up as well as from 6-month follow-up to 12-month follow-up. In the classification that included depression symptoms and positive mental health, 44.8% of the participants changed classification assignment between post-treatment and 6-month follow-up. Most assignments changed to the group *vulnerable* (18.3%, see Table 6). Between 6- and 12-month follow-up, 31.1% participants changed classification assignment; again, most assignments changed to the group *vulnerable* (12.2%, see Table 6).

**Table 6:** Changes between the mental health groups for the subsample (*n* = 328; T2 to T3, T3 to T4).

	T2 ➔ T3	T3 ➔ T4
	% (*n*)	% (*n*)
Depression Symptoms vs. Positive Mental Health		
Complete Mental Health ➔ Troubled	4.3 (14)	1.2 (4)
Complete Mental Health ➔ Vulnerable	16.2 (53)	7.9 (26)
Complete Mental Health ➔ Symptomatic but Content	0.3 (1)	0.3 (1)
Vulnerable ➔ Complete Mental Health	11.9 (39)	8.5 (28)
Vulnerable ➔ Troubled	7.6 (25)	6.4 (21)
Vulnerable ➔ Symptomatic but Content	0	0
Troubled ➔ Complete Mental Health	1.2 (4)	2.1 (7)
Troubled ➔ Vulnerable	1.8 (6)	4.3 (14)
Troubled ➔ Symptomatic but Content	0	0
Symptomatic but Content ➔ Complete Mental Health	0.6 (2)	0.3 (1)
Symptomatic but Content ➔ Troubled	0.6 (2)	0
Symptomatic but Content ➔ Vulnerable	0.3 (1)	0
No Changes	55.2 (181)	68.9 (226)
Anxiety Symptoms vs. Positive Mental Health		
Complete Mental Health ➔ Troubled	1.8 (6)	0
Complete Mental Health ➔ Vulnerable	19.2 (63)	9.1 (30)
Complete Mental Health ➔ Symptomatic but Content	0	0.3 (1)
Vulnerable ➔ Complete Mental Health	13.1 (43)	9.1 (30)
Vulnerable ➔ Troubled	2.7 (9)	4.0 (13)
Vulnerable ➔ Symptomatic but Content	0	0.3 (1)
Troubled ➔ Complete Mental Health	0	1.2 (4)
Troubled ➔ Vulnerable	1.2 (4)	1.5 (5)
Troubled ➔ Symptomatic but Content	0	0
Symptomatic but Content ➔ Complete Mental Health	0.3 (1)	0
Symptomatic but Content ➔ Troubled	0	0
Symptomatic but Content ➔ Vulnerable	0.3 (1)	0
No Changes	61.3 (201)	74.4 (244)

T2 to T4, measurement time points.

In the classification that included anxiety symptoms and positive mental health, 38.7% of participants changed classification assignment between post-treatment and 6-month follow-up. Most assignments changed to the group *vulnerable* (15.2%, see Table 6). Between 6- and 12-month follow-up, 25.6% participants changed classification assignment; again, most assignments changed to the group *vulnerable* (10.7%, see Table 6).

## 4 Discussion

In the present study treatment success was operationalized using the four categories – complete mental health, vulnerable, troubled, symptomatic but content – of the dual-factor model of mental health (3). Regarding the two distinct factors of the dual-factor model the inpatient treatment was effective both in increasing positive mental health and in alleviating psychopathology [cf., (32–35)]. Regarding the assignment to the four groups of mental health over time, most patients had to be classified as *troubled* or *vulnerable* at the pre-treatment assessment. These findings are in line with a previous study on psychiatric inpatients (31). Importantly, not all patients displayed low levels of positive mental health at the pre-treatment assessment: As such up to 14% of patients were classified as completely mentally healthy and up to 2% were classified as symptomatic but content.

The allocation was different at the post-treatment assessment and the follow-up assessments; yet, again there were patients in each quadrant of the dual-factor model. At the post-treatment assessment and at both follow-up assessments most patients (>50%) displayed complete mental health. Still, up to 9.5% were classified as troubled at the post-treatment assessment, and even more so at the follow-up assessment. Symptomatic but content remained a rather rare condition at the post-treatment and the follow-up assessments, whereas many patients were categorized as vulnerable throughout the post-treatment and the follow-up assessments.

In about ten to 20% of the cases, there was an improvement in negative mental health *and* positive mental health between pre- and post-treatment, so that patients were no longer classified as troubled, but as completely mentally healthy [cf., (22, 36)]. In another 10 to 20% of the cases there was a reduction in negative mental health, but no (classificatory relevant) increase in positive mental health between the pre- and post-treatment assessments, so that patients were no longer classified as troubled, but as vulnerable. In 20 to 30%, there was an increase in positive mental health – in patients already displaying low severity of negative mental health – so that patients were no longer classified as vulnerable but as completely mentally healthy. Finally, in about 1% of cases, there was a reduction in negative mental health – in patients already displaying moderate to high levels of positive mental health – so that patients were no longer classified as symptomatic but content but as completely mentally healthy.

These findings support the dual-factor model’s assumption that enhancing positive mental health and alleviating psychopathology do not always co-occur in treatment, and thereby underscore the importance of implementing measures of both constructs in clinical studies (37). To fully evaluate the effectiveness of a therapeutic intervention, both dimensions should be measured. Yet, it is unclear whether a categorization into four groups – as in the present study – or a two-dimensional assessment as used in the studies by Trompetter, Lamers (22) and van Agteren, Ali (36) is more suitable. The latter has the advantage of enabling more complex analysis techniques such as structure equation modelling, which in turn enable more advances modelling approaches such as latent-growth models (SEMs) [e.g., (43)]. However, these methods also require larger sample sizes than available in the present study. The categorization used here has the advantage that it represents a clear and easily understandable outcome measure. At the same time, a categorization does not allow to determine the magnitude of a change in negative and positive mental health. Furthermore, there is no consensus on how to operationalize complete mental health [cf., (44)]; as such very different criteria have been applied in previous studies (3, 6, 30, 31), which means that the comparability of studies is only possible to a very limited extent. With regard to the categorization made here, it has also to be emphasized that the predictive importance of positive mental health for a favorable course of mental disorders is well established (12, 19, 20), whereas there is a lack of studies that have explicitly examined whether complete mental health – as operationalized in the current study – is associated with a greater chance of sustained remission from a mental disorder over longer periods of time. In addition, it must also be clarified whether patients in the *troubled but content* and *vulnerable* categories have a higher risk of relapse and recurrence than patients in the complete mental health category. Only if the categorization is accompanied by real predictive significance, the effort of operationalization appears to be justified [cf., (45)]. This needs to be clarified in future studies. Defining complete mental health would then be the next step [cf., (46)]. Nevertheless, it is already clear that measures of positive mental health should be used as standard outcome measures to describe the effects of interventions on both dimensions of the dual factor model.

There are several limitations to the present study. First, positive mental health was assessed with the 9-item Positive Mental Health Scale (16), instead of a more comprehensive measure of positive mental health. However, the PMH-Scale has been shown to assess various facets of positive mental health (47), and positive mental health as assessed with the PMH-Scale has been shown to be of special importance for positive psychological functioning [e.g., (19, 20, 48)]. Second, because no published norms are available for the PMH-Scale in inpatients a sum score of 14 (on a possible range of 0–27) was chosen as cut-off score [cf., (30, 31)]. Thus, our methodical approach followed available literature on healthy participants [cf., (30, 31)]. Future research should replicate our findings by a person-centered (multilevel) approach. Third, the current study was no randomized-controlled trial, therefore, no statement can be made on the effectiveness of the treatment. Furthermore, it should be kept in mind that this is not a representative sample of mostly depressed inpatients: Nearly all subjects in this study were at least college educated and the treatment took place in a private clinic [in which more affluent people in Germany receive treatment; (49)]. Furthermore, the present study was conducted only on inpatients. Thus, the investigated sample may not represent a research group encompassing all mood disorder patients. Fourth, patients in this study received a very comprehensive range of therapies. However, it is not possible to say which components of the treatment were particularly effective, nor whether all treatment components (including drug treatment) were carried out in full compliance with the treatment guidelines. As this was not an effectiveness study, no specific treatment manuals were prescribed, and treatment adherence was not tracked. Fifth, no information on potential side effects of the medication treatment, participants’ adherence to the treatment, any adjustments made during the treatment, or on medication treatment at the follow-ups was assessed. This lack limits the interpretation of the present findings.

Nonetheless, the findings of the current study underscore the importance of understanding *complete mental health*, as the absence of psychopathology as well as the presence of subjective and psychological well-being. In this sense, complete mental health might be understood as an additional measure of treatment outcome.

## Data availability statement

The raw data supporting the conclusions of this article will be made available by the authors, without undue reservation.

## Ethics statement

The studies involving humans were approved by Ethical Committee of the Faculty of Psychology of the Ruhr-University Bochum. The studies were conducted in accordance with the local legislation and institutional requirements. The participants provided their written informed consent to participate in this study.

## Author contributions

JB: Conceptualization, Data curation, Formal analysis, Resources, Software, Supervision, Validation, Visualization, Writing – original draft, Writing – review & editing. RB: Conceptualization, Data curation, Investigation, Methodology, Project administration, Resources, Software, Supervision, Validation, Writing – original draft, Writing – review & editing. FH: Conceptualization, Data curation, Investigation, Methodology, Project administration, Resources, Software, Supervision, Validation, Writing – review & editing. TT: Conceptualization, Data curation, Formal analysis, Methodology, Project administration, Resources, Software, Supervision, Validation, Visualization, Writing – original draft, Writing – review & editing. GH: Data curation, Validation, Writing – review & editing. JM: Conceptualization, Methodology, Project administration, Resources, Software, Supervision, Validation, Writing – review & editing.
